# Positive effects of the catastrophic Hurricane Patricia on insect communities

**DOI:** 10.1038/s41598-018-33210-7

**Published:** 2018-10-09

**Authors:** Samuel Novais, Luiz Eduardo Macedo-Reis, E. Jacob Cristobal-Peréz, Gumersindo Sánchez-Montoya, Milan Janda, Frederico Neves, Mauricio Quesada

**Affiliations:** 1 Laboratório de Ecologia de Insetos, Departamento de Biologia Geral, Universidade Federal de Minas Gerais, Belo Horizonte, Minas Gerais 31270-901 Brazil; 2 Instituto de Investigaciones en Ecosistemas y Sustentabilidad, Universidad Nacional Autónoma de México, Morelia, Michoacán 58190 Mexico; 3 Laboratorio Nacional de Análisis y Síntesis Ecológica, Escuela Nacional de Estudios Superiores Unidad Morelia, Universidad Nacional Autónoma de México, Morelia, Michoacán 58190 Mexico; 4 Institute of Entomology, Biology Centre CAS, Branisovska 31, 37005 Ceske Budejovice, Czech Republic

## Abstract

Highly seasonal conditions of tropical dry forests determine the temporal patterns of insect abundance. However, density-independent factors such as natural disturbances can abruptly change environmental conditions, affecting insect populations. We address the effects of the Hurricane Patricia (category 5) on species density and abundance of three feeding guilds of herbivorous insects (sap-sucking, folivorous beetles and xylophagous) and predatory beetles associated to the canopy of a tropical dry forest. Hurricane Patricia has been the strongest tropical hurricane ever reported in the Western Hemisphere. Herbivorous insects (sap-sucking and xylophagous) and predatory beetles increased in species density and abundance in the following months after the hurricane, compared to samples before it. The positive response of sap-sucking insects to Hurricane Patricia was probably related to an increase in the availability of new shoots and leaf meristems after the natural coppicing by the hurricane, while xylophagous guild seems to have been positively affected by the increase in the amount and diversity of deadwood resources. The positive response of predatory beetles may be the result of a bottom-up effect due to a greater availability of arthropod preys after the hurricane. We demonstrated that catastrophic hurricane disturbances could be important events that temporarily increase the species density and abundance of insects in tropical dry forests.

## Introduction

Insect communities vary seasonally due to changes in climatic conditions and the availability of food resources^1,2^. Seasonal patterns become more evident when insect abundance and diversity are compared between the dry and wet seasons of highly seasonal environments, such as tropical dry forests^2–9^. In these ecosystems, water stress is a major factor that determines the timing and duration of leafing phenological events regulating plant-insect interactions^3,10,11^. During the dry season, most trees shed their leaves as a drought-resistant mechanism, and then the first rains determine synchronous leaf flushing of most plant species^12,13^. In the case of free-feeding herbivorous insects (e.g. folivorous and sap-suckers), there is a general reduction in abundance and diversity in the dry season^3,6,14^. However, this pattern can vary among insect guilds; for example, xylophagous beetles show increased activity at the transition between wet and dry seasons due to favorable abiotic conditions for flight and selection of new hosts^15^. Temporal patterns for some other insect guilds, such as predatory insects, are often unknown in tropical dry forests.

In addition to variations in certain abiotic conditions among seasons (e.g., dry and wet season) and years (e.g., a drier year compared to a wetter year) in tropical dry forests, density-independent factors such as natural disturbance events can abruptly change resource availability and habitat conditions^16^. Post-disturbance conditions can facilitate the colonization of less competitive species that increase their abundance and expand their distribution^17,18^. Among natural disturbance events, hurricanes play an important role affecting the structure and dynamics of forests around the world^19–26^. Death or architecture simplification of many tree species following hurricanes change abiotic (light, temperature and moisture) and biotic (increase of organic matter in soil, decreased species density) conditions, allowing the growth of pioneer plant species^21,25^. Consequently, structural and compositional changes in plant communities, coupled with an increase in availability of resources (wood debris and foliage regrowth) following hurricanes can have direct and indirect effects on insect communities^27–29^. For example, positive or negative effects on primary consumers can propagate throughout the food chain, causing changes at other trophic levels^30,31^.

Given the difficulty to anticipate the occurrence of such natural disturbances, most studies have attempted to estimate hurricane effects on insect populations comparing abundances among different post-hurricane plots, or after a long period of time have passed between the sampling period before and after hurricane disturbance occurred^28,32–34^. Other studies have attempted to simulate the effect of hurricanes experimentally by trimming tree crowns and comparing arboreal insect abundances before and after manipulations^35,36^. However, these experimental studies could not simulate the effects of a hurricane over larger spatial scales, likely underestimating their effects on insect communities. Plots with experimental tree defoliations are influenced more rapidly by the colonization of insects from nearby intact forests than plots affected by a hurricane^37^. Understanding the effects of hurricane disturbances on forest ecosystem dynamics is of critical importance because the frequency of intense hurricanes (category 4 and 5) is expected to increase in many regions as a consequence of global climate change^38–40^. Therefore, studies that analyze the structure of insect communities before and immediately after hurricanes are needed (but see^41^).

**Figure 1:**
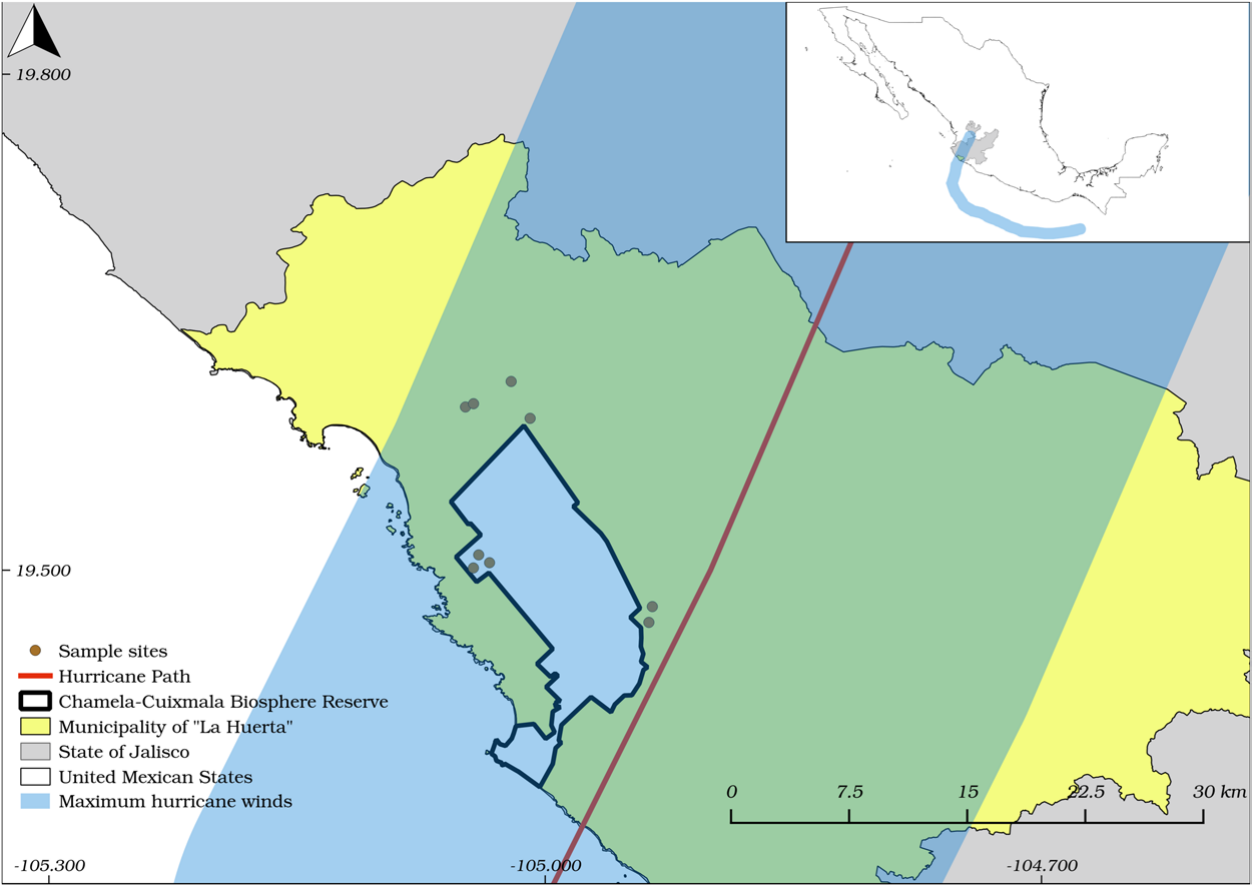
Map of municipality of La Huerta, Jalisco, showing the location of the Chamela-Cuixmala Biosphere Reserve and the nine study sites. The red line and the blue boundaries represent respectively the path of Hurricane Patricia and their maximum winds obtained from historical hurricane tracks of the National Oceanic and Atmospheric Administration (https://coast.noaa.gov/hurricanes/).

On February 2015, we started a study with the objective of evaluate temporal variations in the abundance and species density of three feeding guilds of herbivorous insects (xylophagous, sap-sucking, and folivorous) and predatory beetles in a tropical dry forest canopy. At the end of the rainy season on October 2015, Hurricane Patricia (category 5), the strongest tropical hurricane that has been reported in the Western Hemisphere so far, struck directly on our study sites in Chamela, Jalisco, on the central Pacific coast of Mexico (Fig. 1). Nearly all trees were defoliated, stripped off their branches, snapped, or uprooted by the strong winds, and then the heavy rains associated with the hurricane caused a new vegetative growth^26,42^. The main objective of this study was to evaluate the effects of the catastrophic Hurricane Patricia on different insect guilds in a tropical dry forest canopy, by comparing samples taken before and after the hurricane. We predict that there will be an increase in species density and abundance of insect herbivores following Hurricane Patricia due to foliage regrowth right after the hurricane (sap-sucking and folivorous) and an increase in the availability of dead wood resources (xylophagous). There will also be an increase in species density and abundance of predatory beetles following Hurricane Patricia due to an increased availability of prey. We also evaluated temporal variations in insect communities across different seasons (rainy season, transition and dry season) for three consecutive years, one year before and two years after Hurricane Patricia. Despite the effects of the hurricane, we expect that the strong seasonality of environmental conditions in the tropical dry forest affects the temporal patterns of the insect communities. In addition, we predict that there will be an increase in species density and abundance of free-feeding herbivorous insects (sap-sucking and folivorous beetles) and predatory beetles during the rainy season, and an increase in species density and abundance of xylophagous beetles in the transition between rainy and dry seasons.

## Results

A total of 5,103 insect herbivores was sampled, 2,284 (44.7%) sap-sucking, 116 (2.3%) folivorous beetles and 2,703 (53%) xylophagous. Herbivores were assigned to 275 morphospecies, including 151 sap-sucking, 53 folivorous and 71 xylophagous. For predatory beetles, a total of 1,890 individuals were sampled, assigned to 104 morphospecies (Supplementary Table S1). In terms of number of individuals and morphospecies, Cicadellidae and Scolytinae were the most representative subfamilies/families for sap-sucking and xylophagous guilds, respectively. For folivorous, Chrysomelidae and Curculionidae presented the highest number of individuals, and for predatory beetles Carabidae and Staphylinidae (Supplementary Table S1).

**Figure 2:**
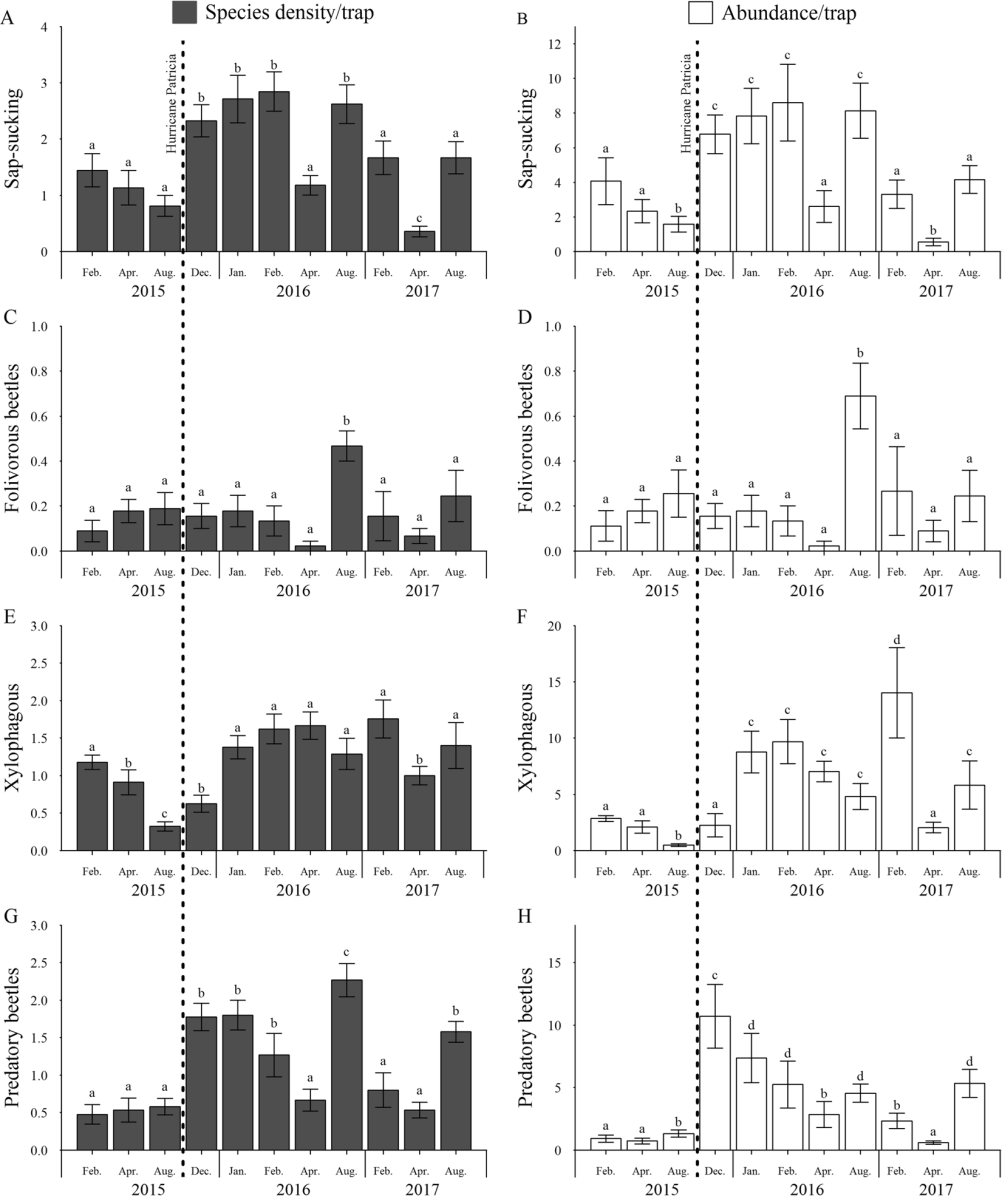
Mean (±SE) species density and abundance of sap-sucking (**A**,**B**), folivorous beetles (**C**,**D**), xylophagous (**E**,**F**) and predatory beetles (**G**,**H**) sampled in eleven periods in the canopy of a tropical dry forest, Jalisco, Mexico. Samplings were carried out in the transition between wet and dry seasons (February), dry season (April) and rainy season (August) of 2015, which represent the periods before the Hurricane Patricia (October 2015). Samplings after the hurricane started in December 2015, followed by January, February, April, and August 2016. In 2017, the samplings were carried out in February, April, and August. Different letters represent significant differences among groups (p < 0.05).

Species density and abundance of all insect guilds varied significantly between sampling periods (Table 1, Fig. 2). However, the seasonal responses varied within guilds and between the years, mainly from the first sampling year (2015) with respect to the following years (2016 and 2017; Fig. 2). The dry season of 2015 was atypical in terms of the rainfall, with a precipitation 1000% greater than expected for this season. Sap-sucking herbivores were more abundant in the atypical dry season compared to the rainy season in 2015. This guild increased approximately 4 fold in abundance and 3 fold in species density after Hurricane Patricia (October 2015) compared to the samples in the previous rainy season (August 2015). In 2016 and 2017, species density and abundance of sap-sucking herbivores were greater in the rainy seasons compared to the dry seasons (Fig. 2A and B). Species density and abundance of folivorous beetles were similar between the atypical dry season and the rainy season of 2015. In the following months after Hurricane Patricia their species density and abundance remained similar to previous samples. In 2016, species density and abundance of folivorous beetles were greater in the rainy season compared to the dry season, whereas for 2017 they were similar between seasons (Fig. 2C and D). Species density and abundance of xylophagous were lower in the rainy season compared to other sampling periods in 2015. This guild increased approximately 3 fold in abundance three months after Hurricane Patricia compared to the samples in 2015, and their species density also increased compared to the samples before the hurricane. In 2016, species density and abundance of xylophagous were similar along the sampling periods, but in 2017 were greater in the transition between rainy and dry seasons (Fig. 2E and F). Predatory beetles were more abundant in the rainy season compared to the atypical dry season in 2015. This guild increased approximately 8 fold in abundance and 3 fold in species density two months after Hurricane Patricia compared to the samples before it. In 2016 and 2017, species density and abundance of predatory beetles were greater in the rainy seasons compared to the dry seasons (Fig. 2G and H).

**Table 1:** Results of Linear Mixed Models showing temporal variation in species density and abundance of herbivorous insects (sap-sucking, folivorous and xylophagous) and predatory beetles sampled in eleven periods in the canopy of a tropical dry forest, Jalisco, Mexico. Collection month was used as explanatory variable. Samplings were carried out in the transition between wet and dry seasons (February), dry season (April) and rainy season (August) of 2015, which represent the periods before the Hurricane Patricia (October 2015). Samplings after the hurricane started in December 2015, followed by January, February, April, and August 2016. In 2017, the samplings were carried out in February, April, and August.

Response variable	AIC (H1)	AIC (H0)	P
Sap-sucking species density	264.58	309.18	<0.0001
Sap-sucking abundance	543.14	572.08	<0.0001
Folivorous beetles species density	−15.43	−6.41	<0.0001
Folivorous beetles abundance	53.56	64.4	<0.0001
Xylophagous species density	171.38	205.56	<0.0001
Xylophagous abundance	612.08	642.31	<0.0001
Predatory beetles species density	168.73	239.97	<0.0001
Predatory beetles abundance	532.23	577.55	<0.0001

## Discussion

Our results showed that catastrophic hurricane disturbances are important density-independent factors that positively affect insect communities in a tropical dry forest. The abrupt changes in forest structure and plant phenology caused by Hurricane Patricia at the end of the rainy season of 2015 lead to an increase in species density and abundance of most studied insect guilds in the following months compared to the samples before the hurricane. Despite this increase, the insect communities oscillated seasonally (i.e. in the following wet and dry seasons) through two years after the hurricane. The arrival of the regular dry season of 2016, for example, caused a reduction in the abundance and species density of most insect guilds. Overall, for 2016 and 2017, the abundance and species density of free-feeding herbivores (sap-sucking and folivorous beetles) and predatory beetles were greater in the rainy seasons, while for xylophagous beetles were greater in the transitions between wet and dry seasons.

A greater species density and abundance of sap-sucking herbivores were found following Hurricane Patricia, compared to the samples before it. Previous studies carried out in a tropical rainforest in Puerto Rico after Hurricane Hugo in 1989 reported a greater abundance of sap-sucking herbivores in post-hurricane gaps compared to closed canopy forests^29,32,33^. The main mechanism attributed to this pattern was related to an increase in the availability of new shoots and leaf meristems due to natural coppicing by the hurricane^43^. Sap-sucking herbivores prefer feeding on these structures because they have increased translocation of nutrients via phloem and xylem vessels^44,45^. In our study, the heavy rains associated with Hurricane Patricia also caused an immediate new vegetative growth of most deciduous plant species, explaining this increase of sap-sucking herbivores. However, the arrival of the regular dry season in 2016 lead to a reduction in species density and abundance of this guild, which increased again in the next rain season of 2016. This seasonal pattern was also consistent between seasons in 2017. These results corroborated previous studies conducted in tropical dry forests which found that sap-sucking populations were severely reduced by stressful conditions in the dry seasons and due to fluctuations in the abundance of new leaves, which are mostly available for them in the rainy seasons^3,6,46^.

Species density and abundance of folivorous beetles did not differ between the samples before and in the following months after Hurricane Patricia. Despite the fact that an increase in the availability of young leaves should lead to an increase in folivorous beetle populations, other changes in abiotic and biotic conditions following Hurricane Patricia may have prevented this from happening. Loss of canopy cover after hurricanes immediately results in increase of temperature and reduction of moisture^47^, which are factors that limit the development, survivorship and fecundity of folivorous beetles^48,49^. For example, Novais and colleagues^50^ demonstrated that folivorous beetles were very sensitive to habitat simplification, and that only a few species were able to remain in habitats with low canopy cover. The quality of foliage regrowth could be another factor negatively affecting folivorous beetle populations; for example, Hunter and Forkner^51^ demonstrated that the regrowth of foliage following Hurricane Opal in United States had higher condensed tannin concentrations, an anti-herbivore defense. Furthermore, the life cycle of folivorous beetles is longer, compared to sap-sucking herbivores, and because most larvae feed and develop on leaves, they could be more susceptible to attack from natural enemies that tend to increase their populations following hurricanes^43,52^, as also observed by our study. For example, Torres^53^ showed an outbreak of 15 species of Lepidoptera caterpillars probably due to a flush of new foliage that occurred after Hurricane Hugo in Puerto Rico, but this outbreak was subsequently controlled by Dipteran and Hymenopteran parasitoids. Similar to sap-sucking herbivores, folivorous beetles also presented a tendency of decrease in species density and abundance with the arrival of the dry season of 2016, and later increase in the rainy season. A tendency to this seasonal pattern was also found in 2017. These results corroborate studies in tropical dry forests for other folivorous insects, such as Lepidoptera caterpillars, which demonstrated that their abundance and species density through the year is seasonal and correlated with rainfall^4,8,54^.

Xylophagous beetles took longer to demonstrate a positive response to post-hurricane conditions compared to sap-sucking and predatory beetles. This delay may be associated with the heavy rains that accompanied the hurricane, disfavoring the colonization of new hosts due to an increase in the moisture content of the available deadwood resources^55^. Later, at the end of the rains, there was an increase in species density and abundance of xylophagous beetles. Strong winds associated with hurricanes cause an increase in the amount and diversity of susceptible deadwood resources, such as tree falls, stem breakage, as well as the fall of coarse and fine wood debris of different tree species, which lead to an increase in xylophagous beetle populations^27,28,56,57^. In addition, standing dying trees, live trees with broken parts or partially uprooted also constitute an important resource for this guild, explaining together their high population levels throughout 2016. During 2017, the xylophagous guild presented clear seasonal variations, with a high abundance in the transition between the previous wet (2016) and the dry season of 2017. These results are in agreement with a previous study conducted in a Brazilian tropical dry forest, which found a greater abundance of Scolytinae beetles at the beginning of a dry season^15^. In our study, a large portion of the xylophagous guild (90% of the individuals, 50% of species) belongs to the Scolytinae subfamily. These beetles spend most of their life cycle protected within the host plant, where the microclimatic conditions are stable and adults only emerge from their brood tree to mate and colonize a new host^55^. During the selection of new hosts, the scolytines are attracted to volatile substances emitted by the plants, usually from recently injured, stressed, dying or dead trees^58–60^, and the new holes usually located at leaf scars, axils, crevices, or other irregularities in the host bark^55^. The flight activity of scolytid adults is usually short, and they seem to avoid very dry and rainy periods to search for a new host, due to the desiccation and the increased moisture content of available deadwood resources, respectively^15,55^. A previous study has shown that the transition between wet and dry seasons may be a more favorable period for beetle flight because air moisture still remains high^15^. In addition, during this period in tropical dry forests most plants begin to lose their leaves due to water limitation, increasing the availability of susceptible stressed hosts and nest entrance sites, such as leaf scars^12^.

Species density and abundance of predatory beetles also increased during the post-hurricane period. This positive response of predatory beetles may be the result of the greater availability of prey after hurricanes, as observed for sap-sucking and xylophagous guilds in our study, and for other groups of invertebrates such as detritivores^29,33^ and dipteran species^41^. However, predatory beetles also decreased in species density and abundance with the arrival of the dry season of 2016, presenting clear seasonal variations with a high species density and abundance in the rainy seasons compared to the dry seasons of 2016 and 2017. In addition to disfavoring abiotic conditions during dry seasons, a decrease in the availability of prey (other arthropods) may be an important factor regulating predatory beetle populations in tropical dry forests^2–6,8^. In our study, species density and abundance of predatory beetles were similar to the pattern presented by sap-sucking and folivorous beetles but differ from that observed for the xylophagous guild. Similarly, a study conducted in an Australian rainforest showed that the seasonality of predatory beetles is more similar to that of folivorous, fungivorous and saprophagous beetles than to xylophagous^61^.

We observed that species density and abundance of most insect guilds presented small variations between seasons in 2015. The El Niño–Southern Oscillation caused changes in the rainfall pattern in the region of our study during the dry season of 2015, with a 1,000% above normal precipitation reported from previous years^62^. Different from expected for the temporal fluctuations in insect communities between seasons in tropical dry forests, 2015 presented small differences, no differences or even a greater species density and abundance of insect guilds during the atypical dry season. An experimental irrigation study conducted in the same tropical dry forest of the present study revealed that at least 200 mm of rainfall during a dry season are needed to cause a full canopy of newly emerged leaves^63^. The heavy rains that occur during the dry season of 2015 exceeded this value, allowing all deciduous trees to recover their canopy, which probably lead to an increase in insect populations.

Our results demonstrated that catastrophic hurricane disturbances could be important events that increase species density and abundance of insects in tropical dry forests. Particularly, the positive effects of the Hurricane Patricia on the studied insect communities were probably associated to the increase in the availability of resources to herbivores (new vegetative growth and wood debris) and prey to predatory beetles. We also demonstrated that despite these positive effects, the insect communities oscillated through the years that followed the hurricane due to the strong seasonality of environmental conditions in the tropical dry forests. In fact, a seasonal pattern congruent to all insect guilds was a lower species density and abundance in the dry seasons, confirming the role of this season in tropical dry forests as an ecological filter that lead to a reduction in insect populations^64^.

## Materials and Methods

### Study area

**Figure 3:**
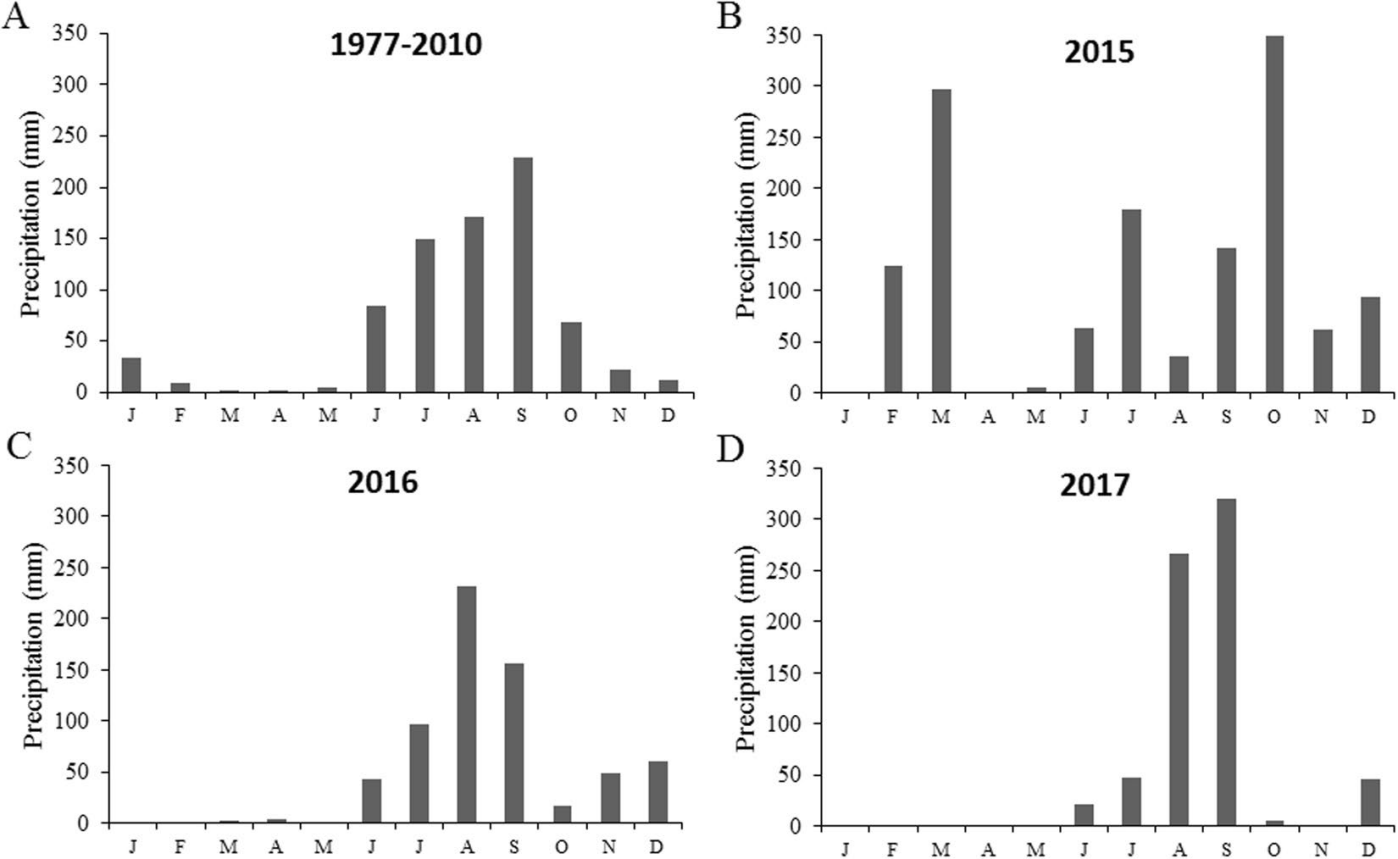
Long-term mean monthly precipitation for the period 1977–2010 (**A**) and total monthly precipitation from January 2015 to December 2017 (**B**–**D**) at Chamela-Cuixmala Biosphere Reserve, in Jalisco, Mexico.

This study was conducted in the Chamela-Cuixmala Biosphere Reserve (CCBR, 19°30′N, 105°03′W) and its surroundings, in Jalisco, Mexico (Fig. 1). The vegetation of the region consists primarily of tropical dry forests with a mean annual rainfall of 748 mm, and a dry season that normally extends from November to May (Fig. 3A^65^).

**Figure 4:**
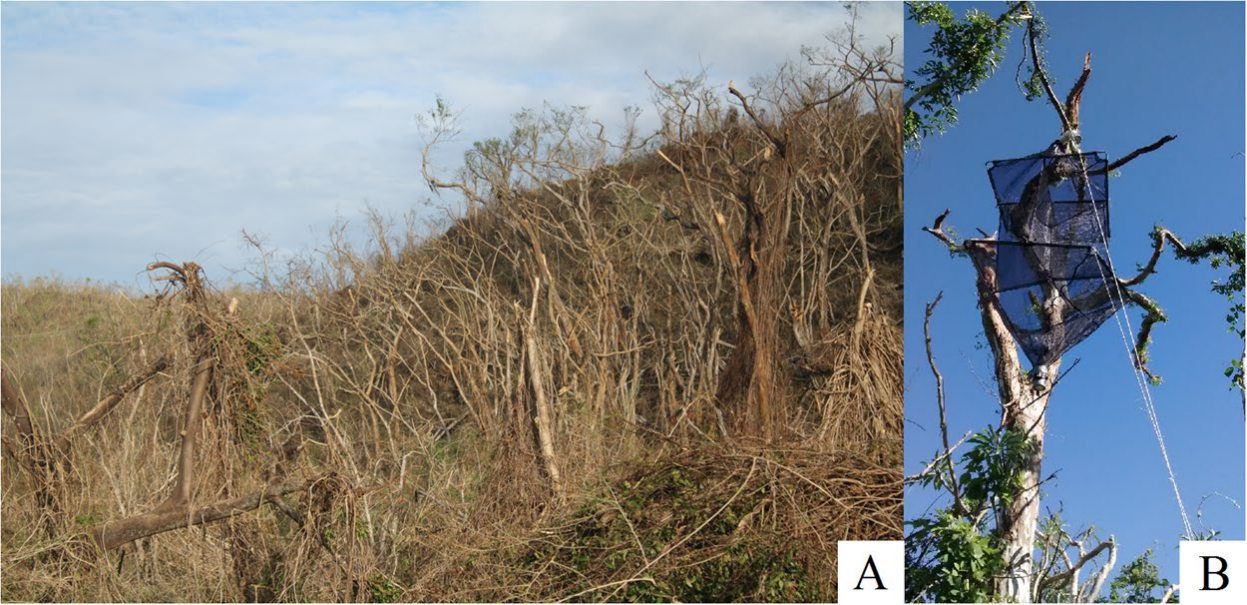
Tropical dry forest in the Chamela-Cuixmala Biosphere Reserve with fallen and denuded trees one week after Hurricane Patricia (October 2015) struck the Chamela region, in Jalisco, México (**A**). Malaise/window trap exposed in the canopy of a tree in January 2016, three months after Hurricane Patricia (**B**). Photos by E. Cristobal-Pérez and S. Novais, respectively.

The year 2015 was atypical in terms of the rainfall by influence of the El Niño–Southern Oscillation^62^. February and March, usually dry months with low rainfall (<10 mm), presented a total rainfall of 116 mm and 282 mm respectively (Fig. 3B). In addition, at 23 of October 2015 Hurricane Patricia, estimated as a category 5 on the Saffir-Simpson Hurricane Wind Scale, reached the southwestern coast of Mexico in the state of Jalisco, near Playa Cuixmala, heavily impacting the Chamela-Cuixmala Biosphere Reserve region (Fig. 1). Heavy rains were associated with the hurricane, for a total of 350 mm in October, extending the rainy season until December (Fig. 3B). Hurricane Patricia is considered as the strongest hurricane that made landfall in the Western Hemisphere which has been reported so far^26^. The village of Chamela was one of the most affected, in which the strong winds tore roofs off of homes and businesses, uprooted, snapped and defoliated nearly all trees (Fig. 4A^26^). On the other hand, the years 2016 and 2017 were normal in relation to rainfall, with well-defined dry and rainy seasons (Fig. 3C and D).

### Sampling design

In 2015, nine 20 m × 50 m plots were selected in CCBR region at a minimum distance of 200 m (Fig. 1), in which the community of insects was sampled eleven times, three before and eight after the Hurricane Patricia (October 2015). Samplings were carried out in the transition between wet and dry seasons (February), dry season (April) and rainy season (August) of 2015, which represent the periods before the hurricane. The insect collection after the hurricane started in December 2015, followed by January, February, April, and August 2016. In 2017, the samplings were carried out in February, April, and August.

### Sampling of insects

We collected the insects using combined flight Malaise/window traps (Fig. 3B^50,66^). With an exception, one trap per plot (9 total) holding two vial collectors with 70% ethanol were exposed for five days (120 h) in the canopy of the same tree during all sampling periods, at a height ranging from 4 to 7 m in the center of each plot. If any of these trees broke after the hurricane, the traps were placed in the canopy of the nearest neighboring tree. August/2015 traps were exposed for 10 days (240 h).

All of the insects belonging to families with prevailing herbivorous habits were considered herbivores^50,67^. The sampled herbivorous insects were grouped into three guilds according to their feeding habit – sap-sucking, folivorous and xylophagous (see^50^). Insects belonging to the suborders Auchenorrhyncha and Sternorrhyncha were considered sap-sucking herbivores, while insects belonging to the suborder Heteroptera were considered herbivores or not depending on the prevailing habit of their respective family. Chrysomelidae, Curculionidae and Megalopodidae were considered folivorous. Butterflies and moths were excluded from the sample. The subfamilies Platypodinae and Scolytinae (Curculionidae) and families Anobiidae and Cerambycidae were included in the guild of xylophagous herbivorous insects, which are groups that preferentially feed on wood parts or build galleries to cultivate fungi for feeding, depending directly or indirectly from the wood resource for survival. Particularly, these xylophagous beetles are attracted to ethanol released by dead and dying trees^59^. Beetles belonging to families with predominantly predatory habits were grouped into the guild of predatory beetles^66^. All insects were identified to family level and morphotyped based on external morphological characters^68^. Species density (number of morphospecies) and abundance (number of individuals) per trap were determined.

The sampled insects were deposited in the entomological collection of the Laboratory of Evolutionary Ecology and Conservation of Tropical Forests of the National Autonomous University of Mexico.

### Statistical analysis

We used Linear Mixed Models (LMER; lme4 package in R) to test whether the abundance and species density of herbivorous insects and predatory beetles were affected by sample period. As suggested by Wardhaugh and colleagues^69^, species density and abundance per trap were chosen over standard rarefaction analysis. To have comparable values among sampling periods we divided species density and abundance per guild (sap-sucking, folivorous, xylophagous and predatory beetles) by the number of days that the traps were exposed in each sampling period. We then used the insect abundance and species density per day trap, as response variables. Collection month was nested within the random effects of the sites sampled during the study^70^. Significance was estimated with an ANOVA between the complete (H1) and the null model (H0). The Akaike Information Criterion (AIC) was used to rank the models, since it represents the uncertainty of the model a lower value of the AIC represents the more parsimonious model. When significant differences were observed between sample periods, contrast analysis was conducted using aggregating levels^71^. If the level of aggregation was not significant and did not alter the deviance explained by the null model, the levels were pooled together (contrast analyses). All analyses were performed using R software version 3.4.1^72^.

## Electronic supplementary material

Supplementary Table S1

## Data Availability

The datasets generated and analysed during the current study are available from the corresponding author upon a request.
