# Supplementary material for: Positive effects of the catastrophic Hurricane Patricia on insect communities
Source: Sci Rep. 2018 Oct 9;8:15042. doi: 10.1038/s41598-018-33210-7 (PMC6177391; doi:10.1038/s41598-018-33210-7)
Supplement: Supplementary file 1 — Supplementary Table S1 [file 41598_2018_33210_MOESM1_ESM.pdf]

Positive effects of the catastrophic Hurricane Patricia on insect communities

Samuel Novais, Luiz Eduardo Macedo-Reis, Edson Cristobal-Peréz, Gumersindo Sánchez-Montoya, Milan Janda, Frederico Neves & Mauricio Quesada

TABLE S1. Morphospecies density (Dens.) and abundance (Abun.) of herbivorous insects (xylophagous, sap-sucking and folivorous) and predatory beetles sampled in eleven periods in the canopy of a tropical dry forest, Jalisco, Mexico. Samplings were carried out in the transition between wet and dry seasons (February), dry season (April) and rainy season (August) of 2015, which represent the periods before the Hurricane Patricia (October 2015). Samplings after the hurricane started in December 2015, followed by January, February, April, and August 2016. In 2017, the samplings were carried out in February, April, and August.

Guild/Taxon	Feb-15		Apr-15		Aug-15		Dec-15		Jan-16		Feb-16		Apr-16		Aug-16		Feb-17		Apr-17		Aug-17		Total	
	Dens	Abun	Dens	Abun	Dens	Abun	Dens	Abun	Dens	Abun	Dens	Abun	Dens	Abun	Dens	Abun	Dens	Abun	Dens	Abun	Dens	Abun	Dens	Abun
Sap-sucking/Hemiptera																								
Auchenorrhyncha																								
Achilidae					4	5	2	4	3	3					3	4					4	6	11	22
Cicadellidae	24	166	24	57	34	122	30	226	39	288	40	322	23	110	37	346	33	143	9	22	25	165	84	1967
Cicadidae					1	1																	1	1
Cixiidae															1	1	1	1			1	2	2	4
Flatidae					1	2									1	1							1	3
Membracidae	1	1							2	2	1	1			1	2					3	3	4	9
Sternorrhyncha																								
Psyllidae	3	7	3	25	3	8	4	6	11	12	7	14	4	5	2	3	1	1			3	3	25	84
Heteroptera																								
Alydidade															1	1							1	1
Coreidae			1	1																			1	1

Cydnidae			2	2			1	2	1	1	1	1	1	1	1	1			1	1	3	3	2	12
Enicocephalidae					1	3	1	1			1	1											1	5
Largidae					1	1	1	4	1	2					3	3					1	1	5	11
Lygaeidae	1	1					2	2			1	1					2	2			1	1	5	7
Miridae	3	7	3	20			3	25	3	44	3	45	1	1	1	3	1	2	1	2	1	1	3	150
Rhopalidae											1	1											1	1
Tingidae	1	1																			1	1	2	2
Not Identified							1	1			1	1			1	1					1	1	2	4
Folivorous/Coleoptera																								
Curculionidae	1	1	1	1	4	6	5	6	4	4	1	1	1	1	8	13	6	12	2	3	3	3	24	51
Chrysomelidae	3	4	6	7	9	10	1	1	3	3	5	5			8	17					8	9	26	56
Megalopodidae					1	7			1	1					1	1							3	9
Predators/Coleoptera																								
Anthicidae					1	1	2	3	3	3	1	3			8	23	1	1	1	1	4	7	10	42
Carabidae	2	17	5	10	3	9	5	249	4	180	3	97	2	36	5	15	2	3	3	4	1	1	9	617
Cleridae			1	1	5	5					1	2			2	5					7	8	11	25
Coccinelidae	2	3	3	4	3	9	4	6	4	18	2	5	2	2	6	26	2	2			3	3	13	78
Histeridae					6	64	1	1			1	1			6	51	1	3	3	3	7	188	11	311
Laemopholeidae									3	6	2	2	1	1	3	3	2	3	1	1	2	3	7	19
Lamparidae					1	7																	1	7
Monotomidae	1	1			1	1	1	2	2	7	2	10	2	20	1	6	2	41	3	8			6	96
Passandridae																	1	1					1	1
Staphylinidae	4	16	6	18	9	23	19	167	17	118	10	224	6	69	18	76	9	51	6	10	14	30	35	694
Xylophagous/Coleoptera																								
Anobiidae			3	6	2	3	1	1	2	3	4	4	3	6	3	9	1	2	2	2	7	36	12	72
Bostrichidae	2	13	2	8	2	2	1	1	1	51	3	39	3	18			4	13	3	3	2	4	5	152

Cerambycidae	1	1			4	4	1	1							3	3	1	1	2	2	9	12	16	25
Curculionidae (Platipodinae)	1	1							2	3	1	2	3	6	2	17	2	2					3	31
Curculionidae (Scolytinae)	10	113	9	80	10	35	8	87	9	337	16	391	15	286	16	187	20	613	15	84	14	210	35	2423
TOTAL	60	353	69	240	106	328	94	796	115	1086	108	1173	67	562	142	818	92	897	52	146	125	701	379	6993
